# Items

**Published:** 1907-10

**Authors:** 


### ITEMS.

The United States Civil Service Commission announces an examination on October 23-24, 1907, to secure eligibles from which to make certification to fill a vacancy in the position of anatomist (male), at $1,600 per annum, in the Army Medical Museum, office of the Surgeon-General, and other similar vacancies as they may occur there.

The Drevet Manufacturing Company, C. H. Marchand, President, of New York, is introducing to the medical profession a concentrated nitrogenous food called Meatox. It is said to possess unusually nutritious properties besides being palatable and very stable. We expect to say something further regarding this product at an early day.

The State Civil Service Commission will hold examinations October 12, 1907, for the following positions among others: Assistant Sanitary Engineer, State Health Department, $1,500; Assistant Steam Engineer, Onondaga County Service, $720 to $900. Library Organiser (woman), $1,200 and $1,500; Sanitary Inspector, State Health Department, $3 to $5 a day; Superintendent, Erie County Lodging House, $1,000 to $1,200; Transit Inspector, Public Service Commission, $1,200; Woman Officer, State Institutions, $300 to $360 and maintenance. The last day for filing applications for these positions is October 5th. For full information address Charles S. Fowler, Chief Examiner, Albany.

---

BLACKWELLS Island (*Daily News*) known the world over as the site of New York's penal institutions, poorhouse and city hospitals, may soon be a thing of the past. It has become too small to acommodate the hordes of petty criminals and miserably poor and sick of the metropolis and larger establishments are proposed somewhere outside of the city, where the prisoners and patients may be made in a measure self-supporting by farm work.

In place of the prisons and poorhouses will be established pleasure places for the teeming millions of the great Eastside. Within another year the great Blackwells Island Bridge over the East river will be opened for use and this will render access to the island easy for the Eastside poor. The proposition is to turn the big prison and almshouse buildings into buildings for pleasure and make the island a popular resort to be owned and conducted entirely by the city.

No city in the world possesses so many pleasure grounds as New York and yet there is no city in the world where so large a proportion of its population is unable to reach these fresh air spots. To Central Park is a 5 cent car fare. To Coney Island is a 25 cent boat ride. To any of the resorts about the city the fare is prohibitive to a majority of the residents of the Eastside, the people who are most shut in and who most need the benefit of a little air that does not come down a narrow light well or up a sewer. Blackwells Island would remedy all of this. A short walk from almost any part of the Eastside would bring it into close connection. Sea bathing could be enjoyed there as fine as anything at Coney. The big buildings could be altered and used for all kinds of settlement work and the humanitarians of New York have become thoroughly aroused to the possibilities which open out to them when the city decides to abandon this island as a place to keep paupers and its criminals.

